# Mechanical Properties of U-Cu Intermetallic Compound Measured by Nanoindentation

**DOI:** 10.3390/ma11112215

**Published:** 2018-11-08

**Authors:** Ruiwen Li, Chuan Mo, Yichuan Liao

**Affiliations:** Institute of Materials, China Academy of Engineering Physics, P.O. Box 9071-13, Mianyang 621907, Sichuan, China; mochuan@caep.cn (C.M.); liaoyichuan@caep.cn (Y.L.)

**Keywords:** uranium intermetallic compound, nanoindentation, modulus, crystal structure

## Abstract

The physico-chemical properties of the Uranium intermetallic compound are of technological importance for improvement of the safety and compatibility of nuclear engineering systems. Diffusion couple samples with U and Cu were assembled and U-Cu intermetallic compounds were fabricated at interface by hot pressure diffusion method at a treatment temperature of 350 °C to 650 °C and at a pressure of 168 MPa in a vacuum furnace. The microstructure and element distribution of the compound phase have been studied by means of SEM, EDS, and XRD. The result showed that a new phase was developed to a thickness of approximately 10 μm with a ration of U:Cu with 1:5. Mechanical properties such as elastic moduli and hardness of the compound have been studied by means of nanoindentation. The nanoindentation testing on sample indicated that hardness of Uranium intermetallic compound are higher than that of metal U and Cu. Uranium intermetallic compound and U have a Young’s moduli with 121 GPa, 160 GPa respectively. The elastic/plastic responses of U-Cu intermetallic compound and U under nanoindentation tests were also discussed in detail.

## 1. Introduction

Preparation and characterization of uranium-based intermetallic compounds have been of concern not only to physical scientists due to a great variety of unusual physical phenomena, but also by engineers in the nuclear realm where U components or fuels have contact with other metals and form an interface layer of intermetallic compounds that will affect the performance of the parts [1,2,3]. Therefore, an understanding of physico-chemical properties of the uranium intermetallic compound is of technological importance for improvement of the safety and compatibility of nuclear engineering systems. Especially, the thickness and properties of the intermetallic compound in the interface layer are important for the evaluation of strength and invalidation of assembly. For example, a relatively thin intermetallic layer may be appropriate for achieving a strong mechanical and chemical bond. However, an intermetallic with greater thickness often acts as a crack initiation site leading to catastrophic failure and poor toughness of the joint.

One of the sources for the formation of U intermetallic compound is usually the presence of U diffusion coupled with metal in engineering where the interface layer size is too small to characterize for a traditional mechanical properties testing method. As we know, nanoindentation was used to probe behavior of the interface layer at a micro- and nano-scale due to its ability to orientate accurately and measure very small loads (1 nN) and displacements (0.1 nm). For example, mechanical properties of binary intermetallic compound of Cu-Sn on the interface were examined by nanoindentation to extract the elastic and plastic properties for evaluation of mechanical behavior and reliability of the solder joint in reference [4,5,6,7,8].

Cu is a good thermal conductor, and when contacted with the U fuel, it can export heat rapidly. In this, U component with Cu can improve the life and safety of components. So, Cu is a good choice in some conditions. The combination of U and Cu will induce an intermetallic compound at interface, the properties of which are not clear at present. Indeed, very few studies have focused on determining the mechanical properties, such as Young’s modulus and hardness, of the U intermetallics [9], while electronics and magnetics properties have attracted much more concern in references [10,11,12,13]. Especially, the mechanical properties of U-Cu alloy have not yet been reported. Furthermore, the intermetallics at the interface and in bulk may differ significantly in terms of density, defects, and crystallographic orientation. In order to accurately model the component behavior, accurate and reliable inputs of physical properties of the intermetallics must be obtained. In this paper, a novel method was used to prepare U-Cu intermetallic compound between U and Cu substrates, and nanoindentation was used to test mechanical properties. Microstructure and concentration in the reaction layer at interface were also analyzed by means of a scanning electron microscope (SEM), Energy Dispersive X-ray Spectroscopy (EDS) and X-ray diffraction (XRD).

## 2. Experimental Procedure

**Figure 1:**
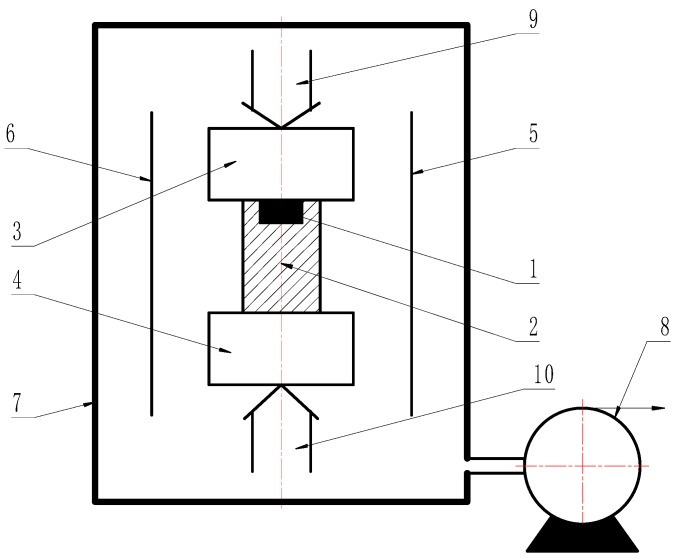
Pressure-thermal treatment vacuum furnace for the couple of U/Cu. 1, U; 2, Cu; 3 and 4, pressure indenter; 5 and 6, heater; 7, furnace body; 8, pumping; 9 and 10 pressure driving

**Figure 2:**
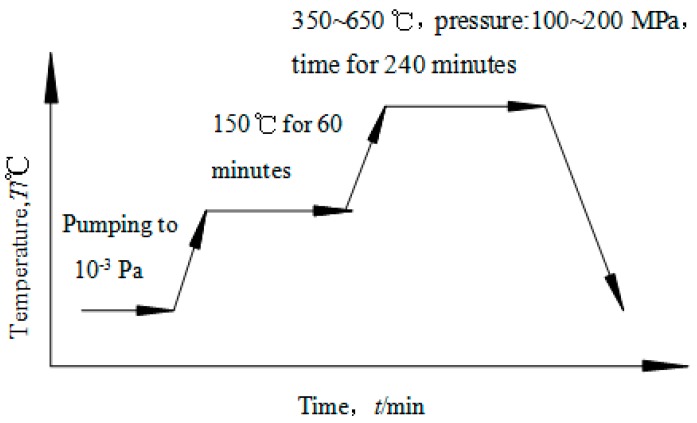
Process for diffusion bonding of U/Cu couple.

Uranium samples (99.998%, from China) were melted by the vacuum induction furnace (VIM, Xinyanindustry, Shanghai, China) and then fabricated into plate used for solid state reaction with Cu. U samples were machined into the dimension of Φ 10 mm × 4 mm and Cu samples (99.999%, from BioRuler) with Φ 20 mm × 30 mm. U and Cu samples were polished by diamond abrasive paper, cleaned by alcohol, washed by ultrasonic, and then dried. Diffusion couples with U and Cu were assembled and put into a vacuum chamber and the orientation in the vacuum chamber was shown in Figure 1. After being evacuated to a vacuum of 8 × 10^−4^ Pa or better, a hot pressing diffusion experiment was immediately carried out at heating temperatures (T) ranging from 350 °C to 650 °C and at pressure of 168 MPa for a holding time (t) from 200 to 240 min after a heat treatment. The sample was then cooled with furnace cooling. The heat treatment process was shown in Figure 2. After the solid-state reaction, the samples were cut from the diffusion bond joints for microstructure and properties observation. A composition profile and morphology of the U-Cu intermetallic compound formed were studied utilizing SEM/EDS with model Noran System SIX (Thermo Fisher Scientific, Waltham, MA, USA) and Laser Scanning Confocal Microscopy (LSCM) OLS4000 (Olympus, Monolith, Japan), respectively. Mechanical properties were investigated by a nanoindention with model TI950 (BRUKER, Hysitron, Minneapolis, MN, USA). The crystal structure was studied at room temperature by means of X-ray diffraction (X’Pert Pro, PANalytical, Eindhoven, Netherlands) using CuKα-radiation, and θ-2θ mode with scanning angle of 20–80°.

## 3. Results and Discussion

### 3.1. Structure and Morphology of U-Cu Intermetallic Compounds

**Figure 3:**
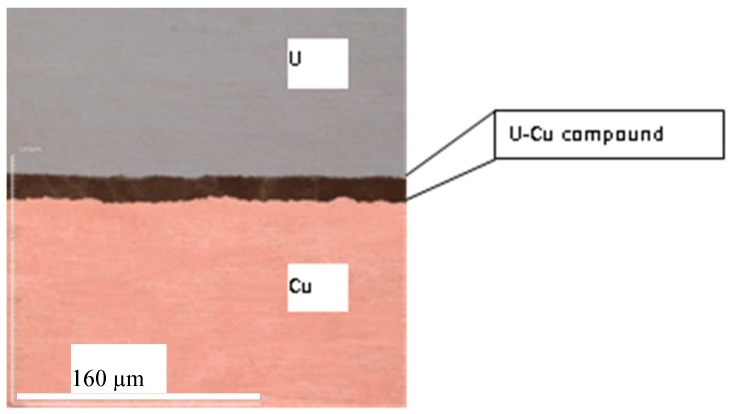
Micrograph of interface at U/Cu couple.

**Figure 4:**
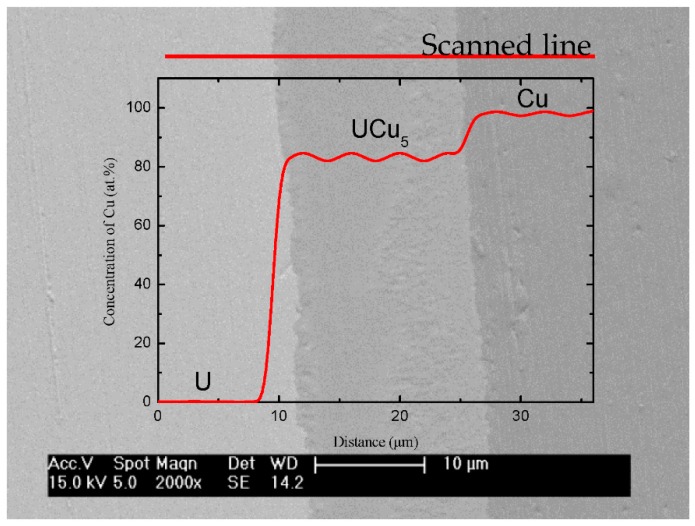
Interface of U/Cu couple: micrograph observation by SEM and element distribution by EDS.

**Figure 5:**
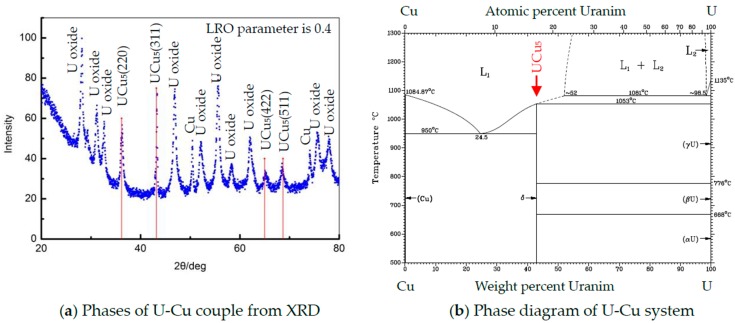
Phase structure of U-Cu system: (**a**) XRD result; (**b**) Phase diagram of U-Cu system.

U-Cu bond samples formed by hot pressure diffusion were then cut and polished to obtain an interface section for analysis. The microstructure of diffusion couples characterized by Light Microscopy was shown in Figure 3, where an interface layer with more than 10 µm thickness formed at the middle between U and Cu substrate. Figure 3 showed a feature of the reaction layer with a different color from that of substrate at U/Cu interface, where a uniform metallograph and color indicated that a thermal stability phase formed under action of temperature and pressure. The metallography shown in Figure 3 and Figure 4 also suggested that no crack was found and bond at interface was very close. To discuss conveniently the phase and structure, an U-Cu phase diagram (from the soft “Binary Alloy Phase Diagrams” (ASM, Russell Township, OH, USA)) was given in Figure 5, which indicated that there is only one intermetallic compound at room temperature. The composition profile analyzed by means of EDS shown in Figure 4 clearly revealed that the reaction production has an invariable ration of U:Cu with 1:5, which indicated that the main phase in the interface layer was a kind of U-Cu intermetallic compound with a stable stoichiometric ratio. Noncontinuous composition change at the interface between intermetallic compound and matrix also indicated that there is no other non-stoichiometric transition or intermediate product formed, but formation of a compound phase. The XRD result, in which the crystallographic planes of UCu_5_ were indicated, shown in Figure 5, and EDS analysis (Figure 4) clearly revealed that the diffusion layer phase was UCu_5_. The compound on interface is very small in length (about 10 um), and diameter of X ray is so big that XRD detecting area include U, Cu, even oxide. So, the signal of XRD is not so good for analysis and refinement. There may be some inaccuracy of cell parameters in this work compared to other references, seen in Table 1. In fact, Table 1 gives the crystallographic data clearly, including space group, atomic positions, and units per cell. It was mentioned that the crystal structure is AuBe_5_ type. It crystallizes in FCC structure with space group F-43m with four formula units (Z = 4). The cell parameters are a = b = c = 7.107 Å under ambient conditions. In the structure, the U atoms occupy one independent position 4a, while Cu atoms are situated in position 4c and 16e. The d_U–U_ (~4.96 Å) in U-Cu intermetallic compound is considerably larger than the Hill limit, and the compound is presumably a narrowing 5f band, which causes even stronger localized character of the 5f electronic states. The density of UCu_5_ is 10.28 g/cm^3^.

### 3.2. Mechanical Properties of U-Cu System at Interface

**Figure 6:**
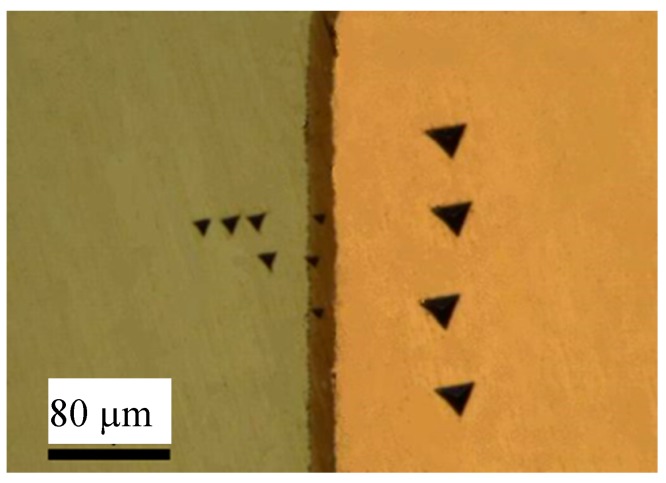
Nanoindentation test on interface of U-Cu.

**Figure 7:**
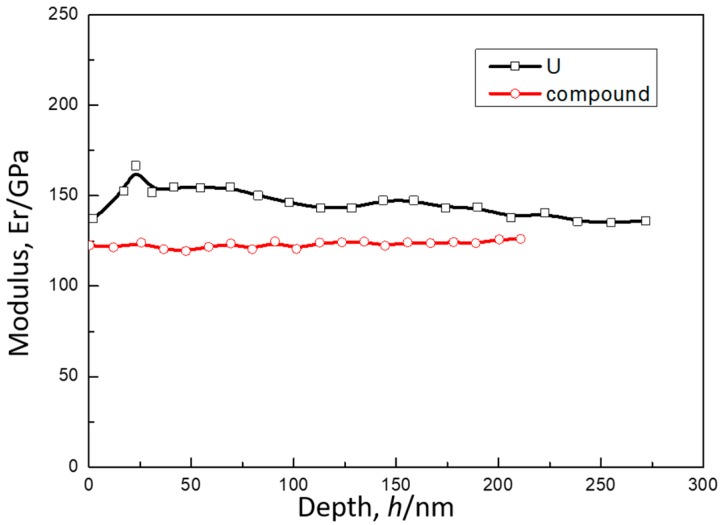
Elastic modulus for U and its intermetallic compound by nanoindentation.

**Figure 8:**
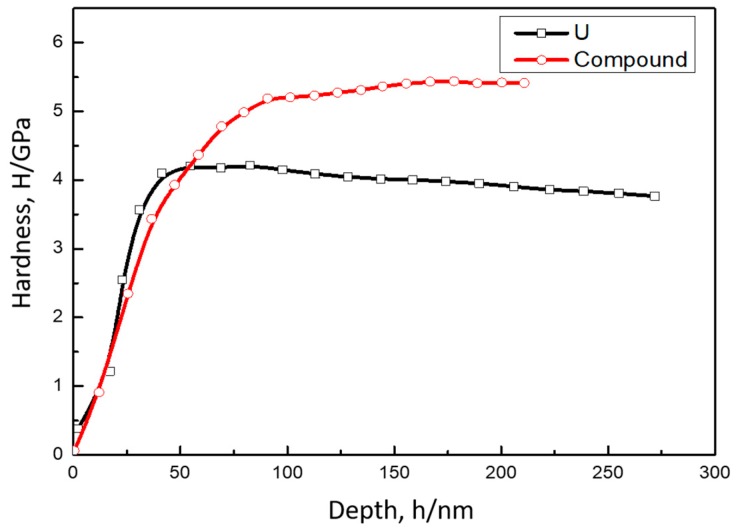
Hardness for U and its intermetallic compound.

Mechanical properties characterization of intermetallic compounds of Uranium and metal elements is necessary for evaluating on compatibility of nuclear engineering systems. The electronic-magnetic properties of U-Cu compounds were widely studied, however, up to authors’ best knowledge, there was no published data of mechanical properties of the U-Cu alloy up to the beginning of this work. In this work, we present nanomechanical properties of U-Cu compound and Uranium obtained by nanoindentation method. U-Cu compound samples used here were obtained by a diffusion couple that was illuminated in Section 2. Nanoindentations were conducted using TI950 with high (shown in Figure 6) and low load (shown in Figure 7) probe, respectively. To avoid substrate affecting the hardness and modulus measurements on U-Cu compound, a Berkovich diamond indenter (BRUKER, Hysitron, Minneapolis, MN, USA) connected with a low load transducer was pressed into interface layer up to 300 nm in depth. The partial-unloading method was adopted to obtain curves of reduced modulus (*E_r_*) and hardness with respect to the indentation depth, which were showed in Figure 7 and Figure 8. By averaging measurement data over indentation depths ranging from 75 to 275 nm, a stable plateau in Young’s modulus (or hardness) was obtained with increasing depth. So, reduced modulus of the U was found to be 146 ± 5.0 GPa, 123 ± 8 GPa for U-Cu compound, shown in Table 2. As we know, the relation between the reduced modulus, *E_r_* and Young’s modulus, *E*, is given by:

(1) $$E_{r}^{-1}=\frac{1-v_{i}^{2}}{E_{i}}+\frac{1-v_{s}^{2}}{E_{s}}\;$$

where, *E* is the Young’s modulus and *v* is Poisson’s ratio, with the subscripts *s* and *i* indicating the sample and the indenter. For the diamond tip employed in this study, *E_i_* = 1141 GPa and *v_i_* = 0.07. By fitting Equation (1) and applying a typical Poisson’s ratio for uranium(*v_s_* = 0.25) [9], the Young’s modulus of Uranium, *E_s_* was calculated as 160 ± 5 GPa. While an assumed value of 0.3 was employed for Poisson’s ratio of UCu_5_ due to lack of the data in literature as Ryan Newell [17] has done, then the Young’s modulus of UCu_5_, *E_s_* was calculated as 121 ± 8 GPa. A comparison of Young’s modulus was made between this work and other’s work like Beeler [18], Wheeler [19], and Yamanaka [9], which indicated that the magnitude of Young’s modulus of U-Cu alloy was not markedly different from other uranium intermetallic compounds and uranium. Indeed, preliminary studies on Young’s modulus of U showed a large degree of variability, the reason for which may be due to difference of preparation and heat pretreatment on the materials or variational measurement way. The discrepancy may also be attributed in part to high anisotropy of the orthorhombic α phase, since small scale indentations are sensitive to local difference in crystallographic direction. Data for Cu were added to Table 2, which were also from nanoindentation.

The values of nanoindentation hardness obtained for U and UCu_5_ were 4.0 and 5.2 GPa, respectively, as given in Table 2. Figure 8 showed hardness dependence on depth which were took as the average of 6 points indents. The hardness for U was close to that determined by Newell [17] as shown in Table 2. But there was no hardness value for U-Cu intermetallic compound found in references to compare. Generally, hardness values for metal intermetallic compounds are higher than that of pure metal, and this law is also correct for the result of U and U-Cu in this paper. The hardness is usually related to the structure of alloy, that is to say if an intermetallic compound with specific stoichiometric ratio is formed, then the hardness will be higher than that of pure metal. For example, UZr_2_ phase with AlB_2_ prototype has a hardness of 5.4 ± 0.20 GPa. But for alloy with solid solution structure, the hardness is determinated by phase of the matrix metal. For example, U-10Mo with a hardness of 4 GPa was corresponded to γ-phase. Actually, large difference in elastic modulus or hardness may cause crack formation within the low-toughness phase. But no crack was observed frequently in the interface of U-Cu system as presented in Figure 3, Figure 4 and Figure 6, which indicated that the compatibility bwtween U, Cu, and U-Cu intermetallic compounds were good in this condition.

**Figure 9:**
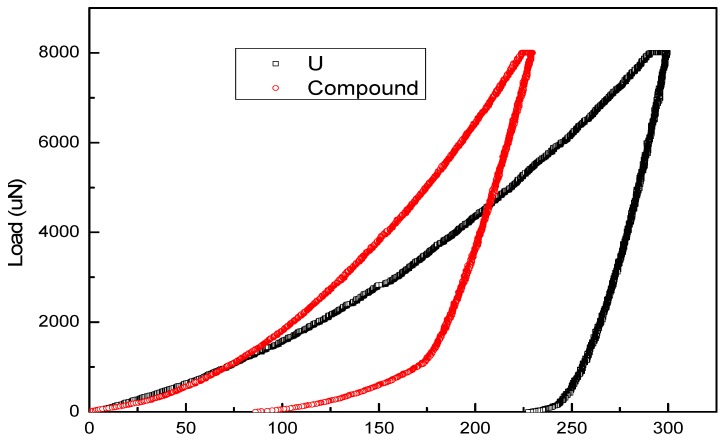
Load-depth curve for U and U-Cu intermetallic compound measured by nanoindentation.

A response with predominant plastic of the two materials can be seen from Figure 9 which showed a typical load-depth curve of both materials, although some elastic recovery with release of the load can also be seen. Meanwhile, elastic recovery for U-Cu intermetallic compound was much larger than that of U, indicating that the former has a decreasing plastic deformation capability. It was also noted that, under the same applied force, the indentation depth for U-Cu intermetallic compound was less than that of U metal showed in Figure 9. This behavior indicated that the hardness of the former was higher than that of the latter, which was consistent with the hardness result shown in Table 2. In both figures a small plateau can be seen at maximum load, which was indicative of creep of the material. The length of plateau for U was larger than that of U-Cu intermetallic compound, which indicated that creep ability of the former was better than the latter. Furthermore, the relative change of the indentation depth at constant load were 2.75% for U, 2.33% for U-Cu intermetallic compound, respectively. The former value agreed well with the data from reference [19]. During indentation, the “pop-in” phenomenon, a sudden jump in displacement due to the transition from elastic to plastic behavior, was occasionally observed, as denoted at a load force of 2830 µN by a circle in Figure 9. However, further work using spherical nanoindentation is required to examine this behaviour in more detail.

## 4. Conclusions

In summary, diffusion couple samples with U and Cu were assembled and U-Cu intermetallic compounds were fabricated at interface by hot pressure diffusion method at a treatment temperature of 350 °C to 650 °C and at a pressure of 168 MPa in a vacuum furnace. The microstructure, element distribution of the compound phase have been studied by means of SEM, EDS and XRD. The result showed that a new phase was developed to a thickness of approximately 10 μm with a ratio of U:Cu with 1:5. Mechanical properties such as elastic moduli and hardness of the compound have been studied for the first time by means of nanoindentation. The result indicated that hardness of Uranium intermetallic compound were higher than that of metal U and Cu. Uranium intermetallic compound and U have a Young’s moduli with 121 GPa, 160 GPa respectively. The elastic-plastic responses of U-Cu intermetallic compound and U under nanoindentation loading were also discussed in detail.

## Figures and Tables

**Table 1:** Crystallographic data of UCu_5_ *.

	UCu_5_
Space group	F-43m
Lattice parameters	a = b = c = 7.107 Å, (7.033–7.038 Å in reference [14]) α = β = γ = 90°
Atomic positions(Wyckoff Positions)	U: 4a Cu1: 4c Cu2: 16e
Density	10.28 g/cm^3^
Formula weight	555.8 g/mol
Units per cell	4

* Some data from references [14,15,16].

**Table 2:** Modulus and hardness of U and U-Cu compound.

Materials	Reduced Modulus/GPa	Young’s Modulus/GPa	Hardness/GPa	Poisson Ratio
U	146 ± 5	160 ± 5	4.0	0.24
U-Cu compound	123 ± 8	121 ± 8	5.2	0.3
Cu	78 ± 10	88 ± 10	1.6	0.34
